# Biological Functions and Molecular Mechanisms of MiR-608 in Cancer

**DOI:** 10.3389/fonc.2022.870983

**Published:** 2022-03-21

**Authors:** Juan Lu, Danhua Zhu, Lanjuan Li

**Affiliations:** State Key Laboratory for Diagnosis and Treatment of Infectious Diseases, National Clinical Research Center for Infectious Diseases, Collaborative Innovation Center for Diagnosis and Treatment of Infectious Diseases, The First Affiliated Hospital, College of Medicine, Zhejiang University, Hangzhou, China

**Keywords:** miR-608, cancer, biomarker, molecular mechanism, tumor suppressor

## Abstract

In recent years, microRNAs (miRNAs) have attracted much attention because of their prominent role in cancer. An increasing number of studies have shown that miRNAs play an important role in a variety of tumors. miR-608 has been reported to be decreased in cancers, especially in solid tumors. miR-608 is regarded as a tumor suppressor, which has been verified through a large number of experiments both *in vivo* and *in vitro*. miR-608 participates in many biological processes, including cell proliferation, invasion, migration, and apoptosis, by inhibiting transmembrane proteins and many signaling pathways. Here, we summarize the expression profile and biological functions and mechanism of miR-608, suggesting that miR-608 is an ideal diagnostic and prognostic biomarker and a treatment target for cancer.

## Background

MicroRNAs (miRNAs) are a class of nonprotein-coding single-stranded RNA with a length of approximately 18-25 nucleotides, and they are encoded by endogenous genes (1–4). miRNAs are highly conserved and tissue-specific (5). miRNAs were first found in Caenorhabditis elegans, and Lee et al. (6) also found that miRNAs participate in *lin-14* gene expression regulation through antisense RNA-RNA interactions. In the past 20 years, the number of miRNA studies has increased substantially. Researchers have shown that miRNAs are involved in the negative posttranscriptional regulation of gene expression and maintain cell homeostasis (7) in the human body by binding with the 3’ untranslated region (3’-UTR) of mRNAs of target genes and degrading the target mRNAs. Generally, a single miRNA has a single mRNA target. However, a miRNAs can possess multiple targets, and a single miRNA target can also be shared by several miRNAs. Proper control of miRNA expression is required for a balanced physiological environment, as these small molecules influence almost every cellular process from the cell cycle and cell proliferation to apoptosis, with a wide range of target genes (8).

In recent research, numerous aberrantly expressed miRNAs were found to be related to the development and prognosis of cancers (9, 10). Among them, miR-608 (GeneID: 693 193), mapped to chromosome 10q24.31, has attracted extensive interest because its dysregulated expression plays a key role in the occurrence and development of various malignant tumors by affecting the posttranscriptional regulation of target genes (11). Further studies have demonstrated that miR-608 expression may affect the treatment efficacy in colorectal cancer (CRC) patients treated with chemotherapy alone or chemoradiotherapy alone (12). Choi et al. (13) demonstrated that miR-608 had the strongest inhibitory effect on the growth of A549 tumor cells by screening a miRNA library. Moreover, the expression level of miR-608 is decreased in many kinds of tumors, including acute myeloid leukaemia (14, 15), bladder cancer (BCa) (11), breast cancer (16), chordoma (17), clear cell renal cell carcinoma (18), gastric cancer (19), glioma (20, 21), melanoma (22), head and neck squamous cell carcinoma (23), hepatocellular carcinoma (HCC) (24, 25), lung cancer (LC) (26–28), osteosarcoma (29), ovarian cancer (30, 31), pancreatic cancer (32), and prostate cancer (33).

In this review, we summarize the latest progress of miR-608 research in the past decade and detail the expression, biogenesis, biological functions, and functional mechanisms of miR-608 in different cancers.

## Regulation of MiR-608 Expression

The 3’-UTR is the crucial area by which miRNAs exert posttranscriptional regulatory functions. Upstream molecules can also bind to the 3’-UTR of miRNAs, downregulate miRNA levels and suppress the biological functions of miRNAs. Generally, upstream molecules mainly include lncRNAs, proteins, circular RNAs (circRNAs), chemical substances and drugs. Among these, 3’-UTR regions of CD44, which is a transmembrane glycoprotein, was firstly identified to bind to miR-608. The CD44 3’-UTR competitively binds with the 3’-UTR of miR-608, thus inhibiting miR-608 functions and releasing the inhibition of downstream mRNAs (34). As additional upstream molecules of miR-608, tumor suppressor candidate-2 pseudogene (TUSC2P) and tumor suppressor candidate 2 (TUSC2) arrest the functions of miR-608 *via* their 3’-UTRs, which subsequently increases translation of TUSC2. TUSC2 is a tumor suppressor, and TUSC2P represses cell invasion, migration, and colony formation *via* the TUSC2P/miR-608/TUSC2 axis (35). Moreover, the TUSC2P/miR-608/TUSC2 axis has been verified to be related to esophageal squamous cell carcinoma (ESCC) (36). In addition, in human lung adenocarcinoma (LUSC), B-cell lymphocyte xL (Bcl-xL), as an anti-apoptotic protein, can interact with hsa-miR-608 and further play a carcinogenic role through the PI3K/AKT, WNT, TGF-b, and ERK signaling pathways (37). Xu et al. (38) revealed that in neuroblastoma, 2,3,7,8-tetrachlorodibenzo-p-dioxin (TCDD) could bind to the aryl hydrocarbon receptor (AhR), induce upregulation of miR-608 and regulate the expression level of cell division cycle 42 (CDC42). An antagonist of AhR, CH223191, can reverse the effect of TCDD, further enhancing the reliability of the above results (38). Kang et al. (39) successfully demonstrated that during Kaposi’s sarcoma-associated herpesvirus (KSHV) lytic infection, open reading frame 57 (ORF57) combined with miRNA and induces the expression of human interleukin 6 (hIL-6), accelerating cell proliferation and tumorigenesis. Thus, the virus can promote the occurrence and development of tumors by interfering with the function of miRNAs (39, 40). Equally notable is that natural products can influence the expression level of miR-608. For example, toosendanin (TSN) upregulates miR-608 and inhibits downstream targets, including Notch1 and Notch2 (41). In addition, circRNAs can also interact with miR-608, and Liu et al. (42) revealed that a circ_0089153/miR-608/EGFR/p53 interaction pathway exists in ameloblastoma (AB). The biological function of circ_0089153 relies on the MAPK signaling pathway (42).

**Figure 1:**
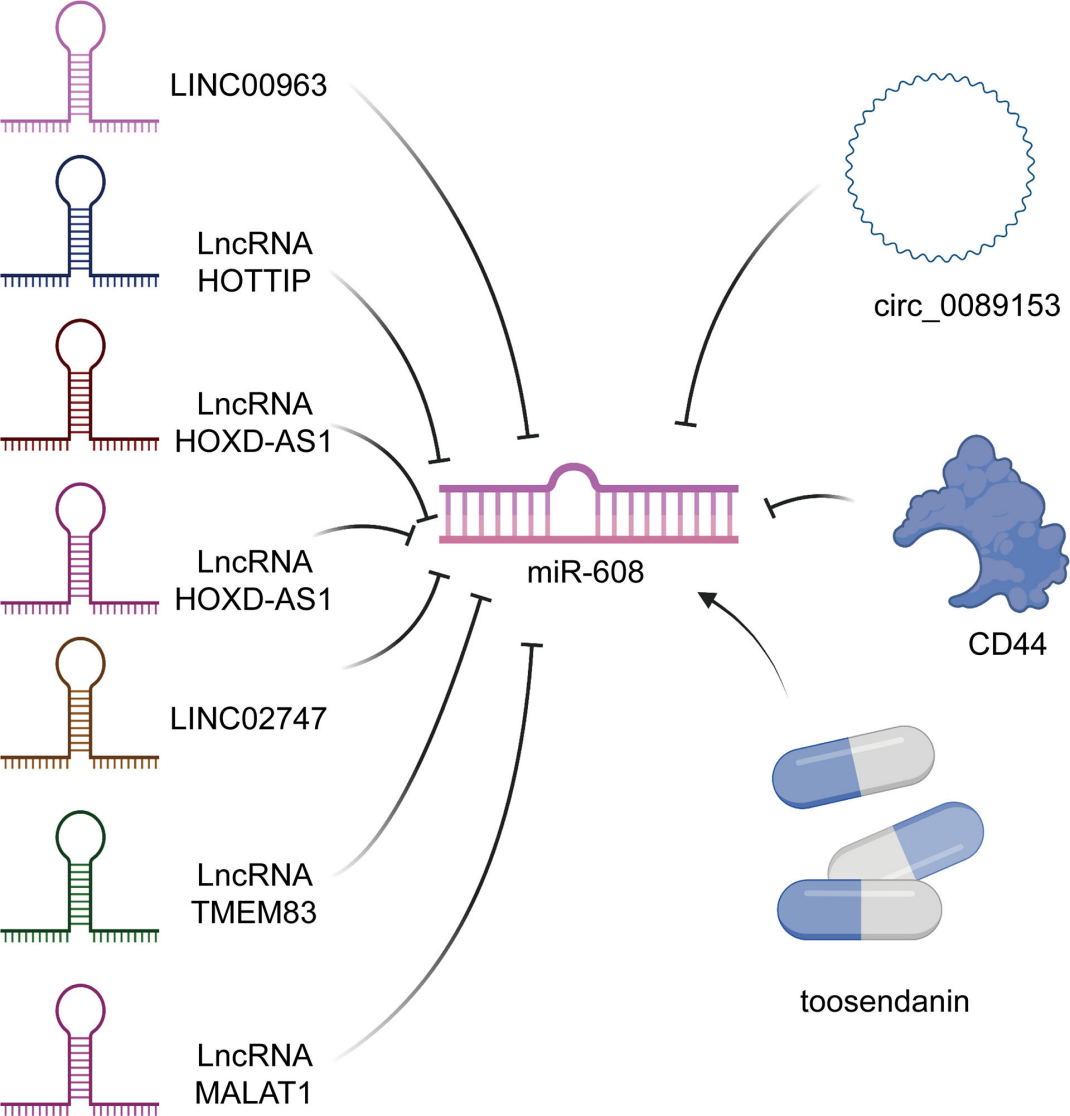
Upstream targets of miR-608. Not only many lncRNAs can regulate the level of miR-608, but also some circRNAs and drugs, such as toosendanin, can also play the role of regulator of miR-608.

Among the confirmed upstream targets of miR-608, long noncoding RNAs (lncRNAs) account for the highest proportion, which will be described in detail below. LINC00963 sponges miR-608 and upregulates the miR-608 target matrix metallopeptidase 15 (MMP-15) (14) in acute myeloid leukaemia (AML). Interestingly, in melanoma, LINC00963 can also interact with miR-608 and further elevate nucleus accumbens associated 1 (NACC1) expression, facilitating cell proliferation, migration and invasion (22), similar to what is seen in AML. Moreover, the lncRNA HOXD-AS1 was also found to bind with miR-608 and promote cell proliferation, migration, invasion, metastasis, and chemoresistance (43). Wang et al. (30) indicated that HOXD-AS1 combines with miR-608 and increases frizzled class receptor 4 (FZD4), participating in the development of ovarian cancer. The lncRNA NORAD has also been found to bind to miR-608 in cancer and upregulate forkhead box O6 (FOXO6) in gastric cancer, accelerating cell growth (19, 44). A similar axis also exists in ovarian cancer, but surprisingly, NORAD induces overexpression of signal transducer and activator of transcription 3 (STAT3) by interacting with miR-608 and functions as a tumor suppressor (31). Remarkably, Zhang et al. (20) also confirmed that lncHAS2-AS1 is another upstream target of miR-608, and STAT1 was found to be an upstream factor of lncHAS2-AS1. Both STAT1 and STAT3 belong to the STAT family. Thus, lncRNAs, miRNAs, mRNAs, and proteins can together form networks of mutual influence and interaction. With the increasing number of relevant studies, a more comprehensive and detailed understanding of these networks will be achieved. In addition, LINC02747, LINC00052, the lncRNA MALAT1, and the lncRNA BLACAT1 can also act as upstream molecules of miR-608 (**Table 1**) (**Figure 1**) (18, 23, 29, 45).

**Table 1:** The upstream and target genes of miR-608 in multiple cancers.

Cancer type	Upstream factor	Target gene	Refs.
Acute myeloid leukaemia	LINC00963	MMP-15	(14)
Acute myeloid leukaemia	LncRNA HOTTIP	DDA1	(15)
Ameloblastoma	circ_0089153	EGFR, p53	(42)
Cancer	TUSC2P and TUSC2	TUSC2	(35)
Cancer	CD44	CDC42	(34)
Cancer	LncRNA HOXD-AS1		(43)
Cancer	LncRNA NORAD		(44)
Clear cell renal cell carcinoma	LINC02747	TFE3	(18)
Esophageal squamous cell carcinoma	TUSC2P	TUSC2	(36)
Gastric cancer	LncRNA NORAD	FOXO6	(19)
Glioblastoma	STAT1/lncHAS2-AS1	PRPS1	(20)
Glioma	toosendanin	Notch1 (Notch2)	(41)
Head and neck squamous cell carcinoma	LncRNA TMEM83	EGFR	(23)
Kaposi’s sarcoma associated with herpesvirus	ORF57	vIL-6, hIL-6	(39)
Kaposi’s sarcoma associated with herpesvirus	ORF57	vIL-6, hIL-6	(40)
Lung adenocarcinoma	Bcl-xL Silencing		
Melanoma	LINC00963	NACC1	(22)
Melanoma	LncRNA MALAT1/LINC00047		(45)
Neuroblastoma	TCDD/AhR	CDC42	(38)
Osteosarcoma	LncRNA BLACAT1	SOX12	(29)
Ovarian cancer	LncRNA HOXD-AS1	FZD4	(30)
Ovarian cancer	LncRNA NORAD	STAT3	(31)

## Dysregulation of MiR-608 in Malignant Diseases

### MiR-608 in Acute Myeloid Leukaemia (AML)

For adults, AML is the most common leukaemia and is characterized by a reduction in normal haematopoietic cells and their replacement by primitive cells. At present, diagnosis is generally achieved by identifying cell immunophenotypes (46–48). Abnormal genetic examination results are recognized as an important prognostic factor. However, accumulating evidence has revealed that some people with normal genetic test results may also have AML (49). Therefore, it is necessary to find new diagnostic markers to screen these patients. Zuo et al. (14) demonstrated that both LINC00963 and MMP15 are upregulated in AML, while miR-608 is reduced. LINC00963 inhibits miR-608 and increases MMP15, which can repress AML cell growth and epithelial to mesenchymal transition (EMT) (14). Interestingly, Zhuang et al. (15) proposed that the lncRNA HOTTIP can upregulate DET1 and DDB1-associated 1 (DDA1) by sponging miR-608. However, the overexpression of DDA1 promotes AML cell proliferation and cell cycle progression (15), which contradicts the research results above. This is because the effector molecules MMP15 and DDA1 have different biological functions. These results remind us that if we want to utilize miRNA as a therapeutic target or diagnostic marker, there may be problems with low specificity.

### MiR-608 in Bladder Cancer (BCa)

BCa is one of the most common cancers of the urinary system. The incidence rate of males is higher than that of females (50). BCa causes approximately 150000 deaths worldwide annually (51). Patients with BCa are often admitted to the hospital as an emergency, and the proportion of patients who are actively found through physical examination is not high. In addition, emergency admission often means poor prognosis (52, 53). Therefore, we urgently need to find new diagnostic biomarkers for the early detection of BCa. Liang et al. (11) found that miR-608 is always downregulated in BCa, which accelerates cell proliferation and cell cycle progression. When miR-608 is upregulated, it inhibits the expression of FLOT1 and induces G1 phase arrest *via* the AKT/FOXO3a signaling pathway. In a xenograft model *in vitro*, upregulated miR-608 was shown to repress BCa cell proliferation (11). In addition, another team also obtained the same results: overexpression of miR-608 can suppress cell survival and invasion and promote cell apoptosis (54). Therefore, miR-608 seems to have the potential to become a diagnostic marker or therapeutic target.

### MiR-608 in Colorectal Cancer (CRC)

CRC currently has the fourth highest incidence rate in the world. In recent decades, with the continuous development of screening technology, the incidence rate of CRC has peaked. Early screening is one of the most effective measures to improve the prognosis of CRC patients, so finding new diagnostic markers remains important (55–57). In the past decade, there have been many meta-analyses and studies of the correlation of miR-608 rs4919510 and CRC, but the conclusions have not been consistent. Kupcinskas et al. (58) revealed that in Europe, miR-608 rs4919510 has no association with CRC. Interestingly, another team proposed that miR-608 rs4919510 is related to the risk of CRC in both African Americans and Caucasians (59). Both Dai et al. (60) and Ying et al. (61) further found that miR-608 rs4919510 is associated with decreased risk of CRC, although Gong’s team disagrees (60–62). In addition, Pardini et al. (63) and Xing et al. (64) discovered that miR-608 rs4919510 is related to the prognosis of CRC, specifically, CRC recurrence-free survival (RFS). The rs4919510 variant G allele of miR-608 may upregulate MRPL43 by causing loss of its function, thus promoting CRC cell proliferation, invasion, and migration, inhibiting cell apoptosis, and ultimately increasing the risk of CRC (65). However, in 2018, another study reported that for the Iranian population, miR-608 rs4919510 was not associated with the incidence rate of CRC but was associated with metastatic risk (66). Therefore, we believe that miR-608 is a potential predictive biomarker of CRC.

### MiR-608 in Hepatocellular Carcinoma (HCC)

HCC is the third leading cancer worldwide. The incidence rate of HCC has been high due to the pervasiveness of hepatitis B virus (HBV) and hepatitis C virus (HCV) infection. Therefore, HCC has caused massive economic costs to human society. It is necessary to find new biomarkers to improve the prognosis of HCC (67–69). Wang et al. (25) found that miR-608 was downregulated in the HCC cell lines HepG2 and SK-Hep-1. Correlation analysis was performed with baseline clinical information. An elevated level of miR-608 was associated with a good prognosis of HCC and was specifically related to tumor size, differentiation, clinical stage, overall survival (OS) and disease-free survival (DFS). Moreover, the researchers also found that miR-608 inhibits its target macrophage migration inhibitory factor (MIF) and promotes cell proliferation (25). Surprisingly, He et al. (24) discovered almost the same pathway, except that the final effector molecule was not MIF but bromodomain-containing 4 (BRD4). This result further confirms that miRNAs can often play a role by targeting multiple targets. If there is synergy between these targets, the specific miRNAs can be considered potential biomarkers. Interestingly, another study revealed that miR-608 rs4919510 is significantly related to good prognosis (long OS) (70). Wang et al. (71) confirmed this conclusion by collecting clinical information from 993 HCC patients and 992 healthy individuals. Therefore, miR-608 has prognostic value and is expected to become a potential therapeutic target for HCC.

### MiR-608 in Lung Cancer (LC)

Among cancers, the incidence rate of LC is the highest in the world, and the incidence rate of LC in women with a history of smoking is the third highest. LC also has the highest mortality among cancers, and the mortality rates in men and women are both the second highest (72–75). Therefore, it is of great significance to reveal the mechanisms underlying the occurrence and development of LC. In 2016, Li et al. (27) discovered that miR-608 rs4919510 was likely associated with both LC risk and susceptibility to LC. In 2019, Xu et al. (26) revealed that compared with that in normal lung tissue, miR-608 expression was downregulated in LC tissue. A dual-luciferase reporter experiment showed that BRD4 was a direct target of miR-608, and the expression level of BRD4 was upregulated in LC tissues. Reduced miR-608 can also promote LC cell proliferation, migration, and invasion through the JAK2/STAT3 signaling pathway (26). These results confirm the results of a study three years ago. Moreover, miR-608 was also found to be downregulated in non−small-cell lung cancer (NSCLC) by of sequencing samples from 106 NSCLC patients and 124 healthy people. Although miR-608 does not affect the incidence of NSCLC, miR-608 can target transcription factor AP-4 (TFAP4) *via* the Hippo-YAP signaling pathway, thereby promoting NSCLC cell apoptosis and inhibiting cell proliferation (28, 76). Through the Hippo-YAP signaling pathway, miR-608 can also target TEA domain transcription factor 2 (TEAD2) and increase cisplatin sensitivity in NSCLC (77). Moreover, miR-608 can exert a tumor-protecting function in small‐cell lung cancer (78). What is more surprising is that in LUSC, miR-608 promotes LUAD cell death and increases the antiproliferative effect of gefitinib *via* the PI3K/AKT, WNT, TGF-β, and ERK signaling pathways (37, 79). Therefore, there is sufficient evidence to indicate that miR-608 is a potential therapeutic target and prognostic biomarker.

### MiR-608 in Pancreatic Cancer

Pancreatic cancer has the worst prognosis of solid tumors (80, 81). Pancreatic cancer mortality ranks fourth among cancers worldwide and has risen considerably in the past few years (82, 83). The incidence of pancreatic cancer continues to slowly increase. This trend is because most pancreatic cancers are exocrine cell tumors, and the prognosis for exocrine cell tumors is worse than that of endocrine cell pancreatic cancers. Of exocrine cell tumors, pancreatic ductal adenocarcinomas (PDACs) are the most common subtype (84). Unfortunately, it is very difficult to detect pancreatic cancer early because of the lack of obvious symptoms. Thus, further discovery of new predictive biomarkers is urgently needed. In 2020, Nishiwada et al. (85) successfully constructed a diagnostic model that consisted of 6 miRNAs and had excellent performance in identifying lymph node metastasis in PDAC patients. The success of this model implies that miRNAs can be very valuable in the early diagnosis of pancreatic cancer. Interestingly, in pancreatic cancer, miR-608 is downregulated, and miR-608 can target ribonucleotide reductase M1 (RRM1) and cytidine deaminase (CDA) and control gemcitabine resistance (32). miR-608 also promotes PDAC cell apoptosis and prolongs PDAC patient OS by binding BRD4 (86, 87) and AKT serine/threonine kinase 2 (AKT2). Therefore, miR-608 has the potential to act as a new diagnostic and prognostic marker and even a treatment target for pancreatic cancer.

### MiR-608 in Esophageal Squamous Cell Carcinoma (ESCC)

The incidence rate and mortality rate of esophageal cancer are among the top ten rates of all cancers (88–90). Esophageal adenocarcinoma (EAC) and ESCC are the two major subtypes of esophageal cancer (88). New biomarkers for ESCC are currently a hot topic of research, and miRNAs have already shown some advantages. Liu et al. (36) revealed that in ESCC EC109 and TE-1 cells, miR-608 targets TUSC2, inhibits cell proliferation and invasion, and promotes cell apoptosis. In addition, miR-608 rs4919510 can also act as a predictive factor for ESCC, as proven by bioinformatics methods (91).

### MiR-608 in Other Cancers

In addition to the cancers mentioned above, miR-608 is also reduced in many other cancers. In chordoma, miR-608 is significantly downregulated and interacts with EGFR and Bcl-xL. The downregulation of miR-608 can accelerate chordoma cell proliferation and migration and repress cell apoptosis (17). In addition, miR-608 sponges RAC2/BCL2L1 and promotes prostate cancer cell proliferation, G2/M transition, and migration (33). In addition, miR-608 also exerts a tumor-inhibiting effect in breast cancer (92), clear cell renal cell carcinoma, gastric cancer, glioblastoma (GBM), glioma, head and neck squamous cell carcinoma, rectal cancer, Kaposi’s sarcoma associated with herpesvirus infection, melanoma, nasopharyngeal carcinoma (NPC), neuroblastoma, osteosarcoma, and ovarian cancer (**Table 2**).

**Table 2:** The expression profile and biological functions and mechanisms of miR-608.

Cancer type	Expression	Clinical features	Target gene	Function	Refs.
Acute myeloid leukaemia	downregulated		MMP-15	cell growth↓ and epithelial to mesenchymal transition (EMT)↓	(14)
Acute myeloid leukaemia	downregulated		DDA1	proliferation↑, cell cycle progression↑	(15)
Bladder cancer	downregulated		FLOT1	proliferation↑	(11)
Bladder cancer	upregulated			proliferation↓, invasion↓, apoptosis↑	(54)
Chordoma	downregulated		EGFR, Bcl-xL	proliferation↑, migration↑, apoptosis↓	(17)
Clear cell renal cell carcinoma	downregulated	high TNM stage and histological grade and poor prognosis	TFE3	proliferation↑	(18)
Colon cancer			NAA10	proliferation↓, migration↓, and cell cycle progression↓, apoptosis↑	(93)
Colorectal cancer			MRPL43	apoptosis↓, proliferation↑, invasion↑, migration↑, cell cycle progression↑	(65)
Colorectal cancer				metastasis↓	(66)
Colorectal cancer					(61)
Colorectal cancer					(58)
Colorectal cancer					(59)
Colorectal cancer					(62)
Colorectal cancer					(63)
Colorectal cancer		recurrence-free survival			(64)
Colorectal cancer					(60)
Metastatic colorectal cancer		tumor recurrence			(94)
Esophageal squamous cell carcinoma					(91)
Esophageal squamous cell carcinoma			TUSC2	proliferation↓, invasion↓, apoptosis↑	(36)
Gastric cancer	downregulated	poor prognosis	FOXO6	cell growth↑	(19)
Gastric cancer					(95)
Glioblastoma	downregulated	poor prognosis	PRPS1	migration↑, invasion↑	(20)
Glioma			Notch1 (Notch2)	apoptosis↑	(41)
Glioma stem cells	downregulated		MIF	proliferation↑, migration↑, invasion↑, apoptosis↓	(21)
Head and neck squamous cell carcinoma	downregulated		EGFR	progression↑	(23)
Head and neck squamous cell carcinoma				tumor growth↓	(96)
Hepatocellular carcinoma		good prognosis, long OS			(70)
Hepatocellular carcinoma					(71)
Hepatocellular carcinoma	downregulated		BRD4	proliferation↑	(24)
Hepatocellular carcinoma	downregulated	tumor size, differentiation, clinical stage, overall survival, disease-free survival	MIF	proliferation↑	(25)
Rectal cancer		better prognosis			(12)
Kaposi’s sarcoma associated with herpesvirus (KSHV)			vIL-6, hIL-6	cell proliferation↑, tumorigenesis↑	(39)
Kaposi’s sarcoma associated with herpesvirus (KSHV)			vIL-6, hIL-6		(40)
Lung adenocarcinoma/non-small-cell lung cancer			AKT2	apoptosis↑	(87)
Lung adenocarcinoma		progression-free survival		anti-proliferation effect of gefitinib↑	(79)
Lung adenocarcinoma				cell death↑	(37)
Lung cancer	downregulated		BRD4	proliferation↑, migration↑, invasion↑	(26)
Lung cancer	downregulated			lung cancer risk↑, susceptibility to lung cancer↑	(27)
Non-small-cell lung cancer	downregulated	does not influence the incidence of NSCLC patients	TFAP4	apoptosis↓, migration↑	(76)
Non-small-cell lung cancer	downregulated		TEAD2	cisplatin sensitivity↓	(77)
Non-small-cell lung cancer	downregulated		TFAP4	apoptosis↓	(28)
Small-cell lung cancer					(78)
Melanoma	downregulated	poor prognosis	NACC1	proliferation↑, migration↑, invasion↑	(22)
Melanoma	downregulated			proliferation↑, migration↑, invasion↑	(45)
Nasopharyngeal carcinoma					(97)
Nasopharyngeal carcinoma					(98)
Neuroblastoma			CDC42		(38)
Osteosarcoma	downregulated		SOX12	proliferation↑, migration↑, invasion↑	(29)
Ovarian cancer	downregulated	poor prognosis	FZD4	proliferation↑, colony formation↑, migration↑, invasion↑	(30)
Ovarian cancer	downregulated		STAT3	cancer growth-inhibiting effects of physcion 8-O-β-glucopyranoside↓, invasion↓, migration↓, apoptosis↑	(31)
Pancreatic cancer	downregulated		RRM1 and CDA	gemcitabine resistance↑	(32)
Pancreatic ductal adenocarcinoma		OS	BRD4	apoptosis↑	(86)
Prostate cancer	downregulated		RAC2/BCL2L1	proliferation↑, G2/M transition↑, migration↑	(33)

↑ means promote and ↓ means inhibit.

### Mechanism by Which MiR-608 Inhibits Tumor Growth

Clinically, in almost all cancers, tumor size is closely related to the prognosis of patients and influences the choice of treatment. Thus, the mechanisms of tumor growth and progression deserve attention. Tumor growth is closely related to the degrees of tumor cell proliferation and apoptosis. Whether a tumor grows often depends on which of these processes is stronger. Increasing evidence shows that miR-608 can significantly inhibit the proliferation of a variety of solid tumors, suggesting that miR-608 is closely related to cell proliferation and apoptosis. Next, we will elaborate the molecular mechanism by which miR-608 is involved in tumor growth from two perspectives.

### MiR-608 and Transmembrane Proteins

Membrane proteins are the main executors of biofilm function. They can effectively participate in cell energy exchange, information recognition and transmission and material transport. According to the different positions of membrane proteins in the cell membrane, these proteins can be divided into peripheral membrane proteins and internal membrane proteins, which are also called transmembrane proteins (99). miR-608 can bind to the 3’-UTR of many transmembrane proteins to inhibit cancer cell proliferation and accelerate cell apoptosis. Among these transmembrane proteins, EGFR is especially important because EGFR can interact with epidermal growth factor (EGF) and induce receptor dimerization and tyrosine autophosphorylation, resulting in cell proliferation. Both Liu et al. (42) and Zhang et al. (17) reported that EGFR is a target of miR-608 and that miR-608 can indirectly attenuate cell proliferation by inhibiting EGFR. Moreover, MMP-15, another transmembrane protein, binds to miR-608 and participates in the progression of AML. Furthermore, rescue experiments indicate that overexpression of LINC00963 promotes cell proliferation and EMT by modulating MMP-15 (14). Interestingly, FZD4 is reported to be upregulated in ovarian cancer, and FZD4 is a transmembrane protein that belongs to the β-catenin signaling pathway. Generally, HOXD4-AS1 exerts tumor-promoting functions through the miR-608/FZD4 axis in ovarian cancer (30). These four studies all clearly indicate that the inhibition of transmembrane proteins by miR-608 leads to suppression of cell growth.

### MiR-608 and Signaling Pathways

miR-608 modulates tumor growth not only by affecting transmembrane proteins but also by affecting multiple signaling pathways. The MAPK pathway has three levels of signal transmission: MAPK, MAPK kinase (MEK or MKK) and kinase of MAPK kinase (MEKK or MKKK). These three kinase levels can be activated in sequence and together regulate a variety of important physiological/pathological effects, such as cell growth and differentiation (100). Importantly, MAPK is also involved in the apoptosis induced by ultraviolet radiation (101). miR-608 targets EGFR and p53 and affects cell cycle processes *via* the MAPK pathway (42). Interestingly, p53 can further activate the PI3K/AKT pathway and influence the cell cycle and mitosis (102). In addition, miR-608 also affects the AKT/FOXO3a signaling pathway to control cell proliferation. miR-608 inhibits both the AKT and FOXO3a kinases and blocks the signaling pathway to attenuate cell proliferation (11) and accelerate cell apoptosis. Moreover, when miR-608 is overexpressed, the expression levels of BRD4, p-JAK2, p-STATA3, CD44, and MMP9 are significantly decreased, indicating that the JAK2/STAT3 signaling pathway is inhibited by miR-608 (26). The inhibition of miR-608 is essential for tumor suppression.

### MiR-608 as a Biomarker

#### MiR-608 as a Diagnostic Biomarker

Early detection and diagnosis are key to improving the prognosis of cancers. An increasing number of studies have shown that the expression of miRNAs is significantly different between cancer tissues and normal tissues (12, 18, 33, 94). This difference can even be detected directly in body fluids (103), which has laid a foundation for the noninvasive detection of miRNA. single-nucleotide polymorphisms of genes encoding miRNAs significantly influence tumor susceptibility and can also act as diagnostic biomarkers for cancers. Ju et al. (18) demonstrated that in clear cell renal cell carcinoma, LINC02747 can sponge miR-608 and further upregulate the mRNA of the target TFE3. The authors suggest that LINC02747 has diagnostic potential for renal cell carcinoma (18). We also believe that miR-608 can be regarded as a diagnostic marker of renal cell tumors because miR-608 is inhibited by upstream LINC02747. In addition, Tokarz et al. (94) found that single-nucleotide polymorphisms of the gene encoding miR-608 can also be used to accurately diagnose metastatic CRC. Moreover, after determining the genotypes of 1358 CRC patients and 1079 healthy controls through sequencing, another team found that miR-608 rs4919510 is obviously related to CRC susceptibility (61). Interestingly, researchers in LC also proposed that miR-608 rs4919510 can significantly affect tumor susceptibility (27).

#### MiR-608 as Prognostic Biomarker

In addition to its diagnostic biomarker potential, miR-608 also has the potential to become a prognostic marker for cancers. Expression of miR-608 is correlated with TNM stage, histological grade, and prognosis; and miR-608 has a close relationship with the prognosis of clear cell renal cell carcinoma (18). In addition, several studies have revealed that miR-608 can function as a prognostic marker (63, 65) and predict CRC recurrence (94). miR-608 rs4919510 was also found to be related to the RFS of CRC (64). Moreover, after collecting basic HCC clinicopathological information, Wang et al. (25) proved that miR-608 is highly correlated with HCC tumor size, differentiation, clinical stage, OS, and DFS. The researchers verified that a decrease in miR-608 facilitated the proliferation of the HCC cell lines HepG2 and SK-Hep-1 (25). Interestingly, Ma et al. (70) confirmed that miR-608 rs4919510 is associated with good prognosis and long OS. To our surprise, miR-608 was reported to be related to the PFS of LUSC patients, and miR-608 expression can indicate poor prognosis of ovarian cancer patients (30, 79). In summary, we found that miR-608 has unprecedented potential for predicting prognosis in solid tumors. However, there are few studies on the prognostic role of miR-608 in haematopoietic system tumors. miR-608 will likely be a promising prognostic marker for multiple tumors, including both solid tumors and non-solid tumors.

#### MiR-608 as Therapeutic Target

Intriguingly, miR-608 has already shown obvious therapeutic effects in tumors according to dozens of studies. TSN can elevate the expression level of miR-608, enhancing glioma cell apoptosis *via* the Notch signaling pathway. *In vivo* experiments also showed that TSN clearly inhibits tumor growth (41). Wang et al. (21) indicated that overexpression of miR-608 attenuates glioma stem cell proliferation, invasion, and migration and induces cell apoptosis, clearly explaining the therapeutic effect of miR-608 in tumors. Moreover, miR-608 can be sponged by LINC00052, regulate the expression of EGFR, and further promote the progression of head and neck squamous cell carcinoma *in vivo* and *in vitro* (23). Overexpression of miR-608 promoted doxorubicin-induced NSCLC cell apoptosis by repressing the expression of TFAP4, and TFAP4 was overexpressed in NSCLC tissues (28). Jiao et al. (22) illustrated that the LINC00963-miR-608-NACC1 pathway might be a potential treatment target for melanoma. Moreover, the roles of the BLACAT1/miR-608/SOX12 axis in osteosarcoma (29), HOXD4-AS1/miR-608/FZD4 axis (30) in ovarian cancer, and lncRNA NORAD/miR-608/STAT3 (31) axis in melanoma indicate that miR-608 could be an ideal therapeutic target. Li et al. (86) revealed that miR-608 can decrease the level of BRD4 and facilitate cell apoptosis. However, in PDAC, miR-608 is usually significantly reduced. A strategy to overexpress miR-608 utilizing gene editing technology or targeted therapy could significantly improve the prognosis for PDAC (86). Zhang et al. (33) also elucidated that miR-608 can obviously alleviate the progression of prostate cancer. Taken together, our findings provide valuable insights for the chemotherapy of multiple tumors, especially solid tumors.

## Conclusions

**Figure 2:**
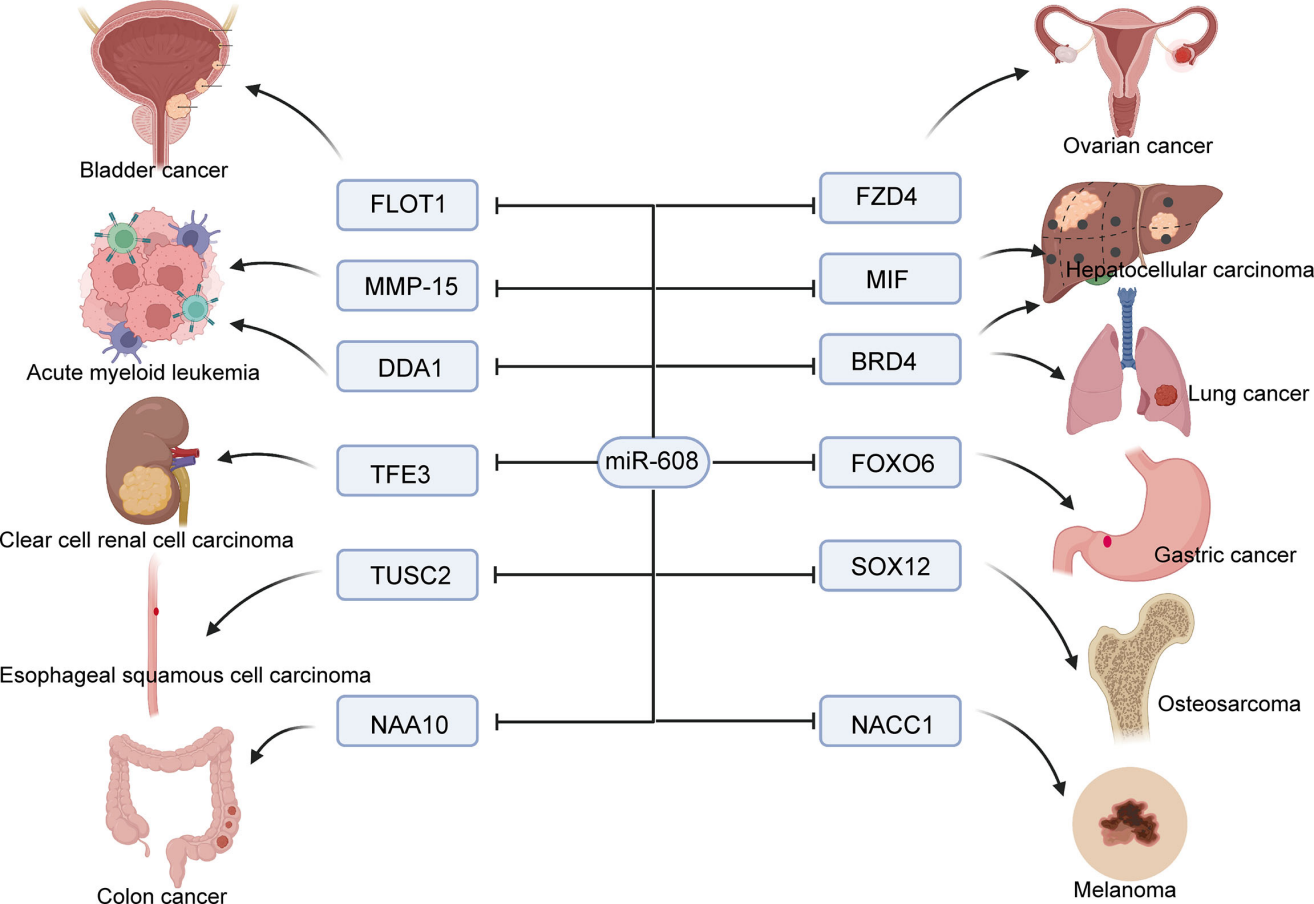
Molecular mechanism of miR-608 affecting tumor cell proliferation. miR-608 targets a large number of genes to inhibit cancer cell growth, including bladder cancer, ovarian cancer, lung cancer, liver cancer, and renal cancer, etc.

In this review, we comprehensively summarized the latest and most valuable research on miR-608. Many researchers in the field of cancer are constantly looking for more potential tumor biomarkers to achieve tumor prevention and treatment. In recent decades, researchers have gradually found that miRNAs play an important role in the occurrence and development of tumors, and an increasing number of people have devoted themselves to studying this field. In addition, miR-608 is a novel miRNA with much potential. miR-608 is decreased in almost all solid tumors except bladder cancer (54). Interestingly, although the results of individual studies are different, miR-608 has been found to consistently play a role in inhibiting cancer in all tumors (**Figure 2**) . This result is surprising and provides new hope for tumor treatment.

Unfortunately, there are no clinical trials related to miR-608 yet, which may be a result of the failure of other drugs with similar targets, such as MRX34, which is miR-34a mimic (104). According to the results of previous clinical trials, the problems with such drugs are probably related to the multiple serious adverse reactions. We speculate that such reactions are caused by the low specificity of miRNA drugs. Thus, in future drug design, organ-specific drug dosages should be designed according to the characteristics of different organs to increase the accuracy of pharmacological effects and reduce complications. In addition, due to the wide distribution of RNases *in vivo*, miRNA drugs also face the challenge of RNA degradation. At present, most strategies use nanocarriers to reduce RNA degradation, but the toxicity of such drugs remains to be studied. In summary, miR-608 has obvious potential for the diagnosis, prognostication and treatment of cancer. To benefit patients in the future, new drugs need to be designed through potential technical routes, and clinical trials need to be carried out as soon as possible.

## Author Contributions

LL designed and guided the study. JL and DZ wrote and edited the manuscript. All authors contributed to the article and approved the submitted version.

## Funding

This work was supported by the National Key Research and Development Program of China (2019YFC0840600 and 2019YFC0840609), and the Independent Project Fund of the State Key Laboratory for Diagnosis and Treatment of Infectious Diseases, the National Key Research and Development Program of China (2016YFC1101404/3).

## Conflict of Interest

The authors declare that the research was conducted in the absence of any commercial or financial relationships that could be construed as a potential conflict of interest.

## Publisher’s Note

All claims expressed in this article are solely those of the authors and do not necessarily represent those of their affiliated organizations, or those of the publisher, the editors and the reviewers. Any product that may be evaluated in this article, or claim that may be made by its manufacturer, is not guaranteed or endorsed by the publisher.
